# Oncogenic KIT-induced aggressive systemic mastocytosis requires SHP2/PTPN11 phosphatase for disease progression in mice

**DOI:** 10.18632/oncotarget.2177

**Published:** 2014-07-07

**Authors:** Namit Sharma, Stephanie Everingham, Li-Fan Zeng, Zhong-Yin Zhang, Reuben Kapur, Andrew W.B. Craig

**Affiliations:** ^1^ Department of Biomedical and Molecular Sciences, Queen's University, Kingston, Ontario, Canada K7L 3N6; ^2^ Division of Cancer Biology and Genetics, Queen's Cancer Research Institute, Kingston, Ontario, Canada K7L 3N6; ^3^ Department of Biochemistry and Molecular Biology, Indiana University, Indianapolis, IN, USA; ^4^ Herman B Wells Center for Pediatric Research, Indiana University School of Medicine, Indianapolis, IN, USA

**Keywords:** aggressive systemic mastocytosis, KIT, SHP2/PTPN11, SHP2 inhibitor

## Abstract

Acquired mutations in KIT are driver mutations in systemic mastocytosis (SM). Here, we tested the role of SHP2/PTPN11 phosphatase in oncogenic KIT signaling using an aggressive SM mouse model. Stable knock-down (KD) of SHP2 led to impaired growth, colony formation, and increased rates of apoptosis in P815 cells. This correlated with defects in signaling to ERK/Bim, Btk, Lyn, and Stat5 pathways in P815-KD cells compared to non-targeting (NT). Retro-orbital injections of P815 NT cells in syngeneic DBA/2 mice resulted in rapid development of aggressive SM within 13-16 days characterized by splenomegaly, extramedullary hematopoiesis, and multifocal liver tumors. In contrast, mice injected with P815 SHP2 KD cells showed less disease burden, including normal spleen weight and cellularity, and significant reductions in mastocytoma cells in spleen, bone marrow, peripheral blood and liver compared to NT controls. Treatment of human mast cell leukemia HMC-1 cells or P815 cells with SHP2 inhibitor II-B08, resulted in reduced colony formation and cell viability. Combining II-B08 with multi-kinase inhibitor Dasatinib showed enhanced efficacy than either inhibitor alone in blocking cell growth pathways and cell viability. Taken together, these results identify SHP2 as a key effector of oncogenic KIT and a therapeutic target in aggressive SM.

## INTRODUCTION

Systemic mastocytosis (SM) is a rare disease characterized by expansion of malignant mast cells in bone marrow, skin, spleen, gastrointestinal tract and liver [1]. Subtypes of SM include indolent SM (ISM), aggressive SM (ASM) and mast cell leukemia (MCL), and these cancers are frequently associated with activating mutations in KIT receptor. For example, the KIT D816V mutation is found in 90% cases of in adults with SM, but is also detected at lower frequency in acute myeloid leukemia (AML) and testicular carcinoma [1-5]. Unlike wild-type KIT that requires binding of its ligand Stem Cell Factor (SCF) for activation, KIT^D816V^ signals in an SCF-independent manner [6]. Studies in mice suggest that the KIT D816V allele is a driver mutation in these cancers since expression of KIT^D816V^, or the analogous mutation in mice (KIT^D814V^), within hematopoietic stem cells (HSCs) leads to myeloproliferative disease (MPD) in mice [7, 8]. Recently, transgenic mice expressing KIT^D814V^ in HSCs were shown to develop an aggressive SM at an early age, while expression in mature mast cells led to similar disease, but with delayed onset [9]. Although KIT inhibitors such as Imatinib are effective for KIT juxtamembrane mutations frequent in gastrointestinal stromal tumors, Imatinib fails to bind and inhibit KIT D816V kinase [10-12]. This has led to identification of more effective inhibitors of KIT^D816V^ kinase activity and SM cell growth though met with limited success probably due to associated mutations in other genes as well [5, 12-16]. Consistently, other broad spectrum kinase inhibitors such as Dasatinib show promise in vitro, but have yielded disappointing results for treating SM patients [17]. Consistent with evidence with other targeted therapies, it is evident that resistance to KIT inhibitors develops via activation of downstream pathways driven by Ras, Lyn, Btk and Stat5 [18-20]. With the identification of other druggable targets within the oncogenic KIT pathway, it is hoped that new combination therapies may prove more effective in treating SM.

SHP2 (also called PTPN11) is a tyrosine phosphatase that participates in a diverse array of signaling pathways, and is critical for survival of trophoblast stem cells, HSCs, metabolic control, cardiac and muscle development, and connective tissue mast cell homeostasis [21-25]. SHP2 is composed of two SH2 domains, a PTP domain, and a C-terminal domain with two sites of tyrosine phosphorylation. The activity of SHP2 is tightly controlled by intramolecular interactions between N-SH2 and PTP domains, which can be overcome by SH2 interactions with tyrosine phosphorylated ligands [26]. In mast cells, SCF-induced KIT activation leads to recruitment of SHP2 to the juxtamembrane domain of KIT via the adaptor protein Gab2 [27]. In bone marrow-derived mast cells, SHP2 promotes KIT signaling to cell survival and proliferation pathways [22, 27]. Recent studies have also implicated SHP2 in playing a key role in oncogenic KIT signaling in a variety of cell types. In *spi-1* transgenic mice, leukemic proerythroblasts with KIT^D814Y^ (or D818Y) signal via SHP2 to enhance cell survival in vitro and tumor growth *in vivo* [28, 29]. In both erythroblast and mast cell leukemia cell lines, SHP2 silencing led to reduced Ras/MEK/ERK pathway activation, upregulation of Bim, and apoptosis [28, 29], which was consistent with our results in SHP2 knock-out (KO) mast cells [22]. In a KIT^D814V^-driven MPD model, SHP2 KO impaired transformation of bone marrow progenitors, and a small molecule inhibitor of SHP2 (II-B08) [30] was shown to synergize with a PI3K inhibitor to repress mast cell leukemia in MPD mice [31]. Together, these studies identify SHP2 as a key mediator of wild-type KIT and oncogenic KIT signaling pathways. Given the frequency of KIT mutations in SM, further testing of SHP2 as a druggable target is certainly warranted for this disease.

Here, we report that SHP2 silencing in P815 mouse mastocytoma cell line harboring KIT^D814Y^ mutation results in impaired signaling to ERK, Btk, Lyn and STAT5 pathways, and reduced rates of cell growth and colony formation. SHP2 knock-down (KD) cells were also more susceptible to apoptosis induced by KIT inhibitor treatment, and showed reduced Bim phosphorylation. In syngeneic mice injected with P815 control or SHP2 KD cells, the development of aggressive SM disease in bone marrow, spleen and liver was significantly reduced with SHP2 silencing. SHP2 inhibitor II-B08, when combined with Dasatinib, prevented oncogenic KIT signaling and cell growth in human and mouse mastocytoma models *in vitro*.

## RESULTS

### SHP2 promotes proliferation and colony formation in KIT^D814Y^ driven mastocytoma cells

**Figure 1:**
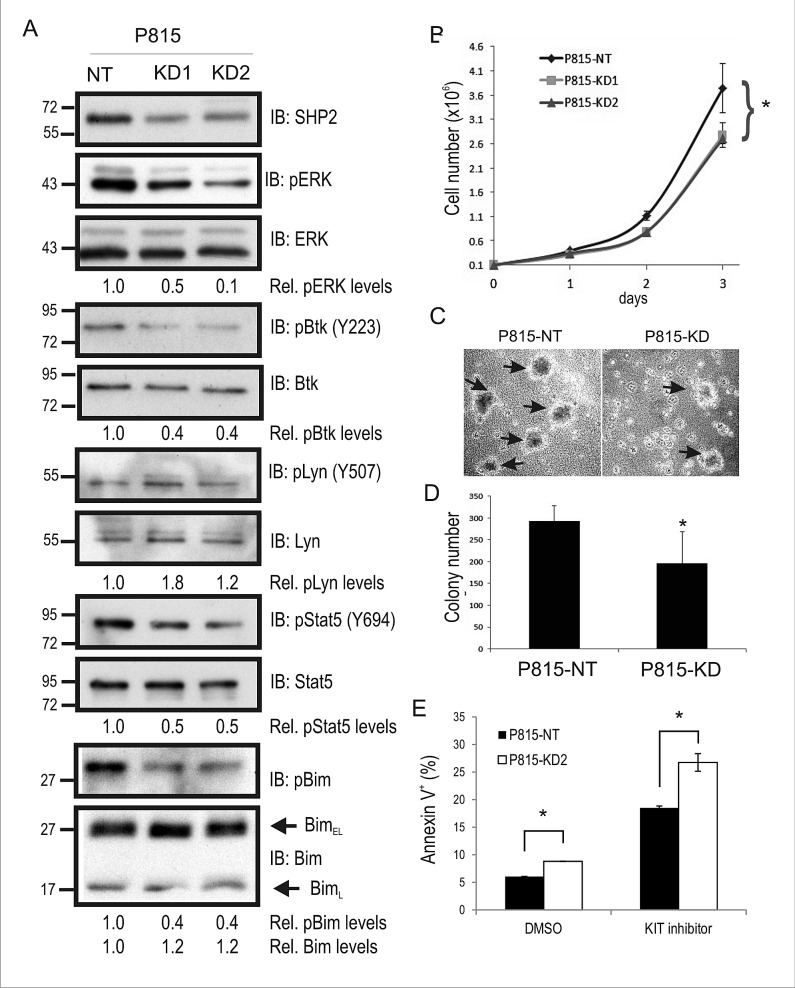
SHP2 promotes mouse mastocytoma cell growth, and colony formation A. Lysates from P815 cells transduced with non-targeting (NT) control shRNA (P815-NT), SHP2 shRNA1 (P815-KD1), and SHP2 shRNA2 (P815-KD2) were subjected to IB with SHP2, pERK, ERK, pY223-Btk, Btk, pY507-Lyn, Lyn, pY694-Stat5, Stat5, p-Bim, and Bim antisera. Densitometry values are shown below panels for relative phosphorylation levels (phospho-specific/total) or protein levels (Bim/ERK). Positions of relative mass markers in kilodalton are shown on left. B. P815-NT, -KD1 and –KD2 were seeded in triplicate wells (1×10^5^) on day 0, and counted daily until day 3. Graph depicts the average cell number (mean ± SD; triplicate samples) for each time point (* indicates significant difference between NT and KD1 or KD2 cells, p <0.05). C. Representative phase contrast images for P815 colonies (shown by arrows) formed in semisolid methylcellulose medium at day 8 for P815-NT and P815-KD cells. D. Graph depicts average colony numbers (mean ± SD) for P815-NT and P815-KD cells in methyl cellulose medium at day 8 (triplicate samples; * indicates significant difference between NT and KD, p <0.05). E. P815-NT and P815-KD cells were treated with vehicle (DMSO) or KIT inhibitor (SU11652, 250 nM) for 18 hours prior to AnnexinV staining and analysis by flow cytometry. Graph depicts percent Annexin V positive cells (mean ± SD; triplicate samples; * indicates significant difference between NT and KD2 cells, p <0.05). All results are representative of at least 3 separate experiments.

To study the function of SHP2 in KIT-driven SM, we transduced P815 mouse mastocytoma cell line that expresses KIT^D814Y^, with either a non-targeting (NT) shRNA or two separate shRNAs against mouse SHP2. Puromycin resistant cells were pooled together, and SHP2 levels were assessed by immunoblot (IB). Compared to P815-NT cells, we observed a 50-60% reduction in SHP2 levels in KD1 and KD2 cell lysates (Fig. 1A; ERK served as a loading control). To test whether SHP2 KD altered key pathways downstream of KIT^D814Y^, IB analyses were performed using a panel of phospho-specific and control antibodies for several key mediators of oncogenic KIT signaling. SHP2 KD cells showed reduced phosphorylation of ERK compared to NT control cells (Fig. 1A), and this is consistent with SHP2 silencing in other KIT-driven cancer models [28, 29, 31]. We also observed a similar reduction in Btk autophosphorylation (Y223) in SHP2 KD cells and increased phosphorylation of the C-terminal inhibitory site (Y507) in Lyn kinase (Fig. 1A). Interestingly, both Lyn and Btk are implicated in cell growth and survival in human SM models [18]. Increased Lyn pY507 in SHP2 KD cells is consistent with SHP2-dependent dephosphorylation of this inhibitory site downstream of the G-CSF receptor [32], and oncogenic KIT receptor. We also examined Stat5 phosphorylation since it is linked to oncogenic KIT signaling [12], and SHP2 promotes Stat5 activation downstream of FLT3-ITD in AML cells [33]. Indeed, Stat5 phosphorylation was reduced in SHP2 KD cells compared to control (Fig. 1A). Defects in ERK activation in a variety SHP2-deficient cell models leads to reductions in phosphorylation and proteosomal degradation of the proapoptotic Bim protein [22, 25, 29]. In P815-KD cells, we also observed reduced Bim phosphorylation and increased Bim_EL_ levels compared to NT control (Fig. 1A). Together, these results implicate SHP2 in promoting signaling to growth and survival pathways in P815 mastocytoma cells harboring the KIT^D814Y^ driver mutation.

The effects of SHP2 silencing on P815 cell proliferation was assessed in 2D cell culture conditions and colony assays. Both P815-KD1 and -KD2 cell pools showed a significant defect in cell growth rates compared to P815-NT cells over a 3 day period (Fig. 1B). Since both SHP2 shRNAs caused comparable defects in signaling and growth rates, we conclude that this is due to SHP2 downregulation and not off-target effects. Subsequent assays were performed using a single P815-KD cell pool (P815-KD2), which was most efficient in maintaining SHP2 silencing with multiple cell passages (data not shown). To test effects of SHP2 silencing on the malignant phenotype of P815 cells, we measured mastocytoma colony formation in semi-solid, methylcellulose media in the absence of exogenous cytokines or colony-stimulating factors. Within 8 days of plating in methylcellulose we observed increased colony formation for P815-NT cells compared to P815-KD cells (Fig. 1C). Quantification of these results from multiple experiments revealed a significant reduction in mastocytoma colony formation with SHP2 silencing (Fig. 1D). Together, these results identify SHP2 as a positive regulator of KIT^D814Y^-driven mastocytoma growth in 2D and 3D culture conditions.

Since SHP2 promotes survival signaling from wild-type and oncogenic KIT [22, 25, 29], we tested for effects of SHP2 silencing on KIT-dependent survival of P815 cells using Annexin V staining and flow cytometry. There was a small but significant increase in apoptotic P815-KD cells compared to P815-NT cells with vehicle alone (DMSO), and a more substantial increase following treatment with a low dose (250 nM) of KIT inhibitor SU11652 for 18 hours (Fig. 1E). These results support a role for SHP2 in promoting survival signaling downstream of oncogenic KIT receptor.

### SHP2 silencing impairs development of aggressive SM in mice

**Figure 2:**
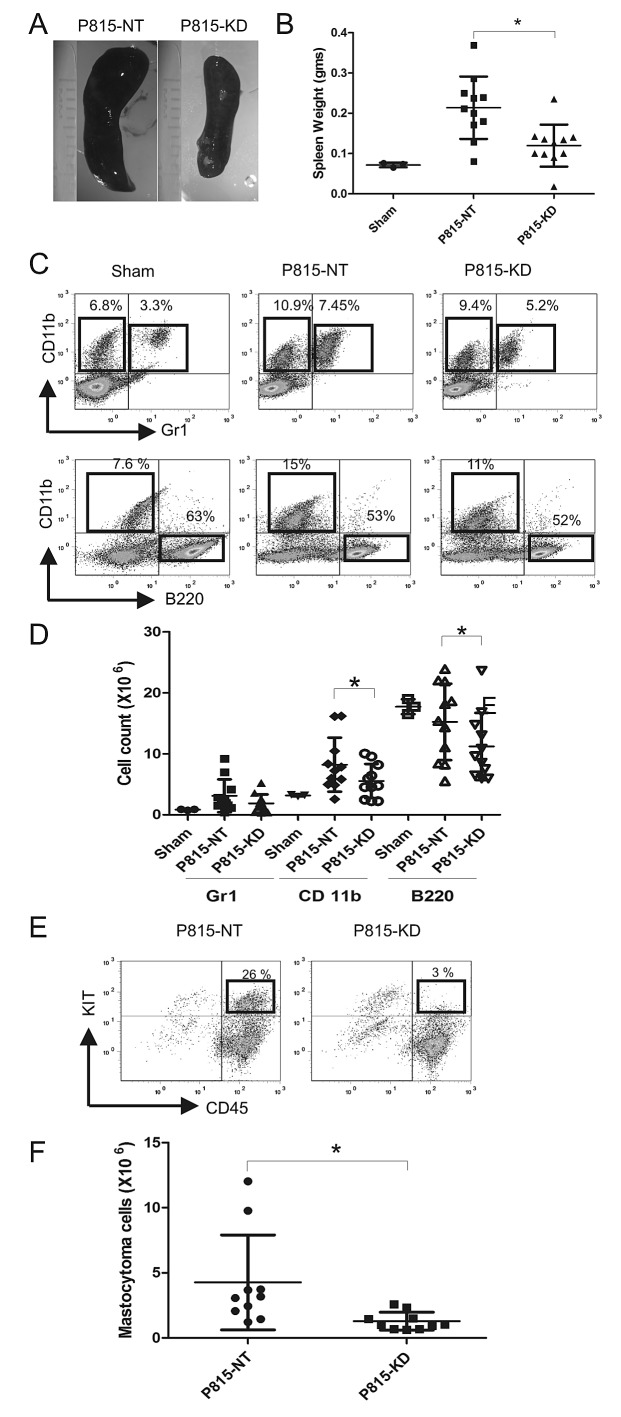
SHP2 silencing prevents splenomegaly in a mouse model of SM A. Representative images of spleens isolated from DBA/2 mice at day 13 following retro-orbital injection of saline (sham), or P815-NT cells, or P815-KD cells (5 × 10^3^ in 100 μl; 3-4 mice/group; 3 experiments). B. Graph depicts spleen weights from saline-injected sham mice (n=3), or mice injected with P815-NT or P815–KD cells (n=11) at day 13 (mean ± SD; * indicates significant difference between groups, p <0.05). C. Splenocytes isolated from mice described above were analyzed by flow cytometry. Representative histograms are shown for splenocytes stained with FITC-Gr1/PE-CD11b/PEcy5-B220, with mean values shown for single and double positive populations (n=3; results are representative of 3 independent experiments). D. Graph depicts absolute cell numbers (x10^6^) for Gr1^+^, CD11b^+^, and B220^+^ cells in spleen from mice described above (* indicates significant difference between NT and KD, p <0.05). E. Representative histograms are shown for splenocytes stained with PE-KIT/PEcy5-CD45, with mastocytoma cells identified as double positives, from mice described above. F. Graph depicts the absolute mastocytoma cell numbers in spleen (mean ± SD; n=10; * indicates significant difference between NT and KD, p <0.05).

To test the role of SHP2 in progression of aggressive SM *in vivo*, we employed a previously described model involving injection of P815 cells in retro-orbital sinus of syngeneic DBA/2 mice [34]. In this model, a rapid and fatal expansion of SM occurs within weeks, characterized by extensive infiltration of bone marrow (BM), spleen, blood and liver, leading to splenomegaly and liver damage [34]. We performed retro-orbital injections of DBA/2 mice with saline (sham), P815-NT or P815-KD cells. Mice receiving P815-NT cells appeared moribund within 13 days, whereas those injected with P815-KD cells or saline showed no overt phenotypes (data not shown). Mice from all groups were sacrificed on day 13, and peripheral blood and tissues harvested to monitor development of SM. All mice injected with P815-NT cells developed splenomegaly, whereas those injected with P815-KD cells had only modest spleen enlargement compared to sham controls (Fig. 2A, B). To analyze the effects on splenic cellularity, single cell suspensions from spleens were stained with antibodies to detect neutrophils (Gr1^+^/ CD11b^+^), macrophages (CD11b^+^Gr1^−^), B-lymphocytes (CD11b^−^B220^+^), and mastocytoma cells (CD45^+^KIT^+^). Subsequent analyses by flow cytometry revealed that compared to sham controls, mice from P815-NT and P815-KD groups showed increased populations of neutrophils and monocytes, and reduced B lymphocytes (Fig. 2C). Since spleen size and cellularity were also different between groups, we compared absolute cell numbers, and observed a pronounced increase in neutrophils and monocytes in mice injected with P815-NT cells compared to sham (Fig. 2D). In contrast, only a modest rise in these populations was observed for P815-KD group compared to sham (Fig. 2D). Both P815-NT and P815-KD groups showed a trend towards reduced B220^+^ cells compared to sham controls (Fig. 2D). Mastocytoma cells were also detected in spleen by flow cytometry, with a significant reduction in the P815-KD group compared to P815-NT (Fig. 2E, F). These results demonstrate that SHP2 silencing lessens development of splenomegaly in mice subjected to a model of aggressive SM.

**Figure 3:**
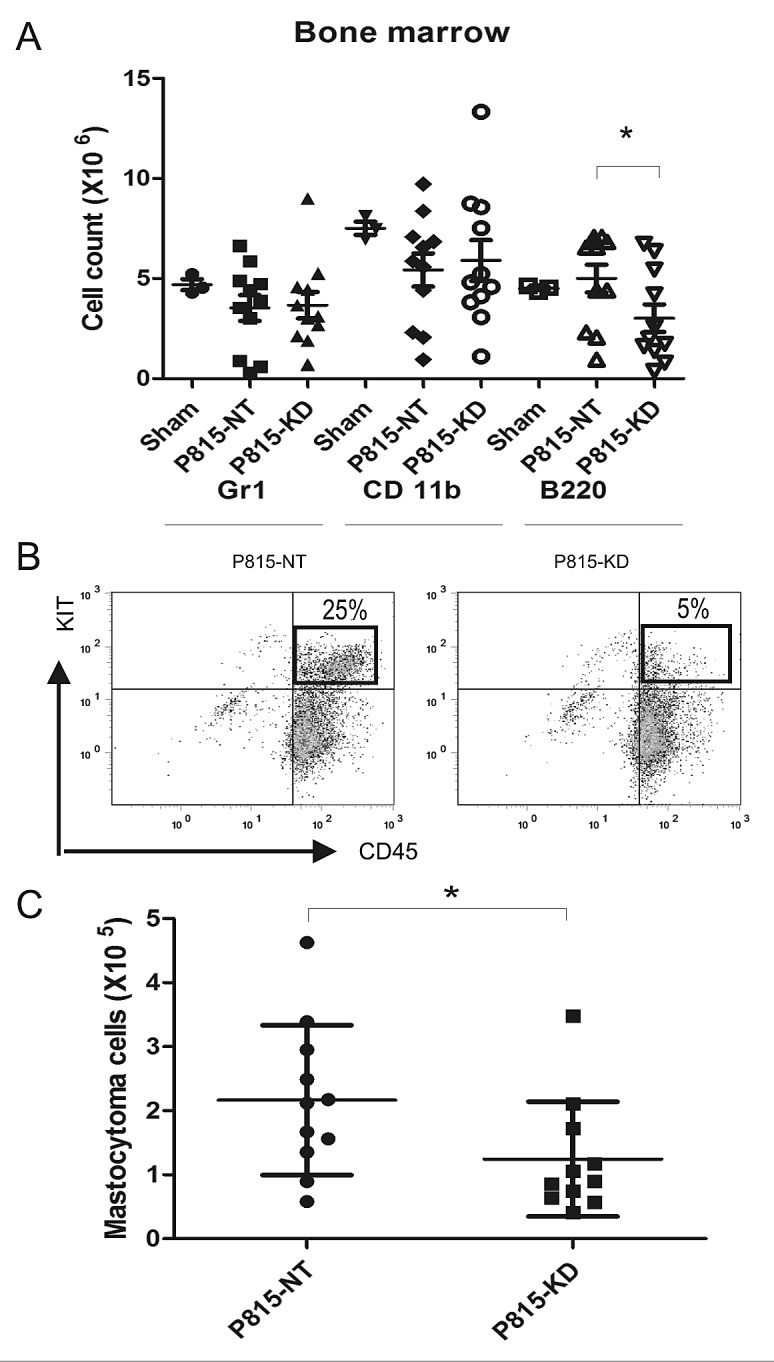
SHP2 silencing limits mastocytoma infiltration of bone marrow in a mouse model of SM A. BM cells were isolated from DBA/2 mice at day 13 following retro-orbital injection of saline (sham), or P815-NT cells, or P815-KD cells. BM cells were stained with FITC-Gr1/PE-CD11b/PEcy5-B220 and analyzed by flow cytometry. Graph depicts absolute cell numbers (x10^6^) for Gr1^+^, CD11b^+^, and B220^+^ cells in BM from sham (n=3), or mice injected with P815-NT or P815-KD cells (mean ± SD, n=11; * indicates significant difference between NT and KD, p <0.05). B. Representative histograms are shown for BM cells stained with PE-KIT/PEcy5-CD45, with mastocytoma cells identified as double positives, from mice described above. C. Graph depicts the absolute mastocytoma cell numbers in BM (mean ± SD; n=11; * indicates significant difference between NT and KD, p <0.05).

Since BM infiltration was previously reported in this SM model [34], we next analysed BM cellularity for the sham, P815 NT and P815 KD groups. Compared to sham mice, both P815-NT and P815-KD groups showed reductions in immature myeloid cells (CD11b), neutrophils (Gr1) and B cell progenitors (B220; Fig. 3A). This correlated with a substantial population of mastocytoma cells in BM for the P815-NT group, and to a lesser extent for the P815-KD group (Fig. 3B; 25% and 5%, respectively). Quantification of the mastocytoma burden in BM for multiple experiments revealed a significant reduction with SHP2 silencing (Fig. 3C). To verify that SHP2 silencing was maintained in vivo, mastocytoma cells were isolated from BM by selection with puromycin, and were shown by IB to have retained silencing of SHP2 (data not shown).

**Figure 4:**
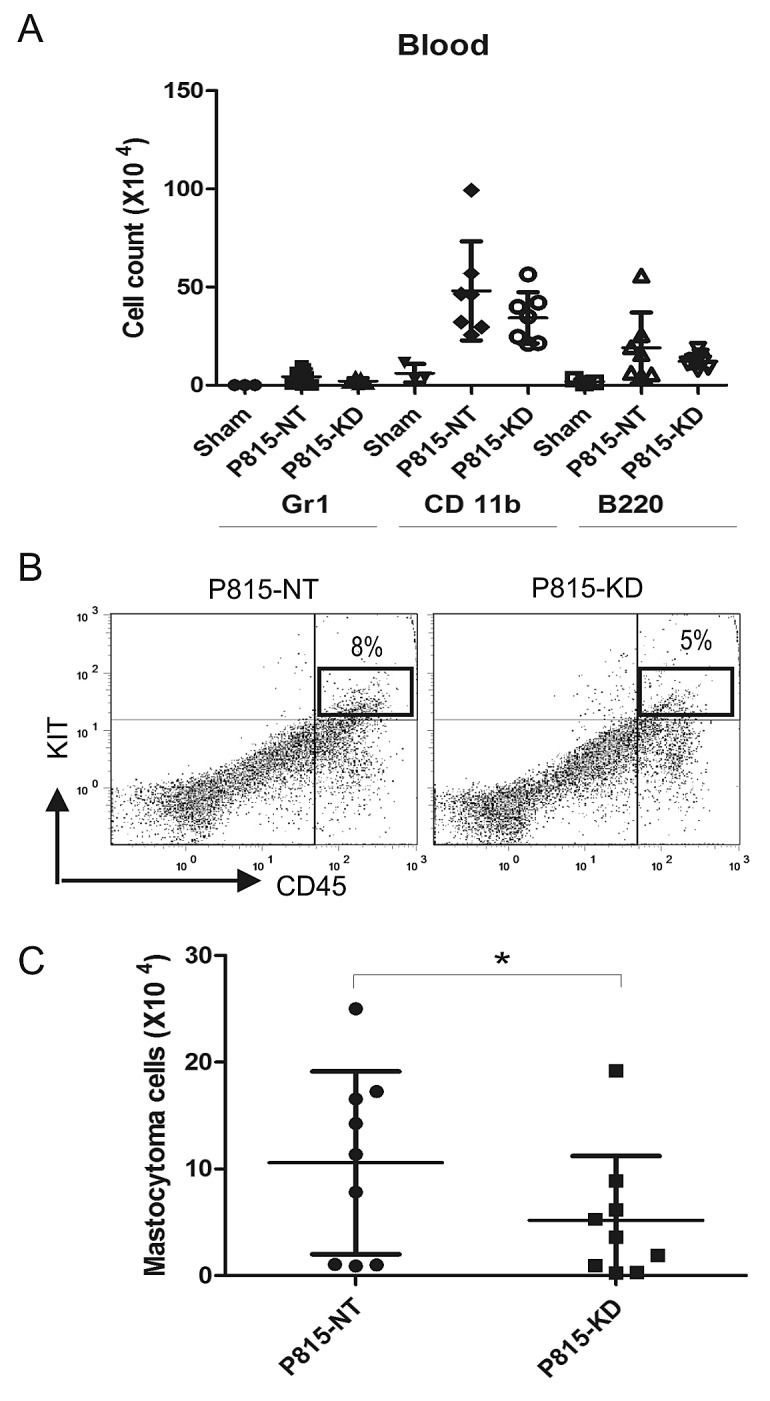
SHP2 silencing reduces mastocytoma burden in peripheral blood A. Single cell suspensions from peripheral blood were isolated from DBA/2 mice at day 13 following retro-orbital injection of saline (sham), or P815-NT cells, or P815-KD cells. Following lysis of erythrocytes, cells were stained with FITC-Gr1/PE-CD11b/PEcy5-B220, and analyzed by flow cytometry. Graph depicts absolute cell numbers (x10^6^) for Gr1^+^, CD11b^+^, and B220^+^ cells in peripheral blood from sham (n=3), or mice injected with P815-NT or P815-KD cells (mean ± SD, n=9 from 3 separate experiments). B. Representative histograms are shown for peripheral blood cells stained with PE-KIT/PEcy5-CD45, with mastocytoma cells identified as double positives, from mice analyzed in 3 independent experiments as described above. C. Graph depicts the absolute mastocytoma cell numbers in peripheral blood (mean ± SD; n=9 from 3 separate experiments; * indicates significant difference between NT and KD, p <0.05).

To assess the systemic spread of mastocytoma in DBA/2 mice injected with P815-NT or P815-KD cells, peripheral blood was collected at day 13. After erythrocyte lysis, peripheral blood neutrophils, monocytes, B cells and mastocytoma cells were analyzed by flow cytometry. Compared to sham, both P815-NT and P815-KD groups showed elevated levels of monocytes (Fig. 4A). Although no significant differences were associated with circulating immune cells in P815-KD group, we did observe a significant reduction in mastocytoma burden (Fig. 4B, C). Together, these results are consistent with a protective effect of SHP2 silencing on the progression of SM in mice.

**Figure 5:**
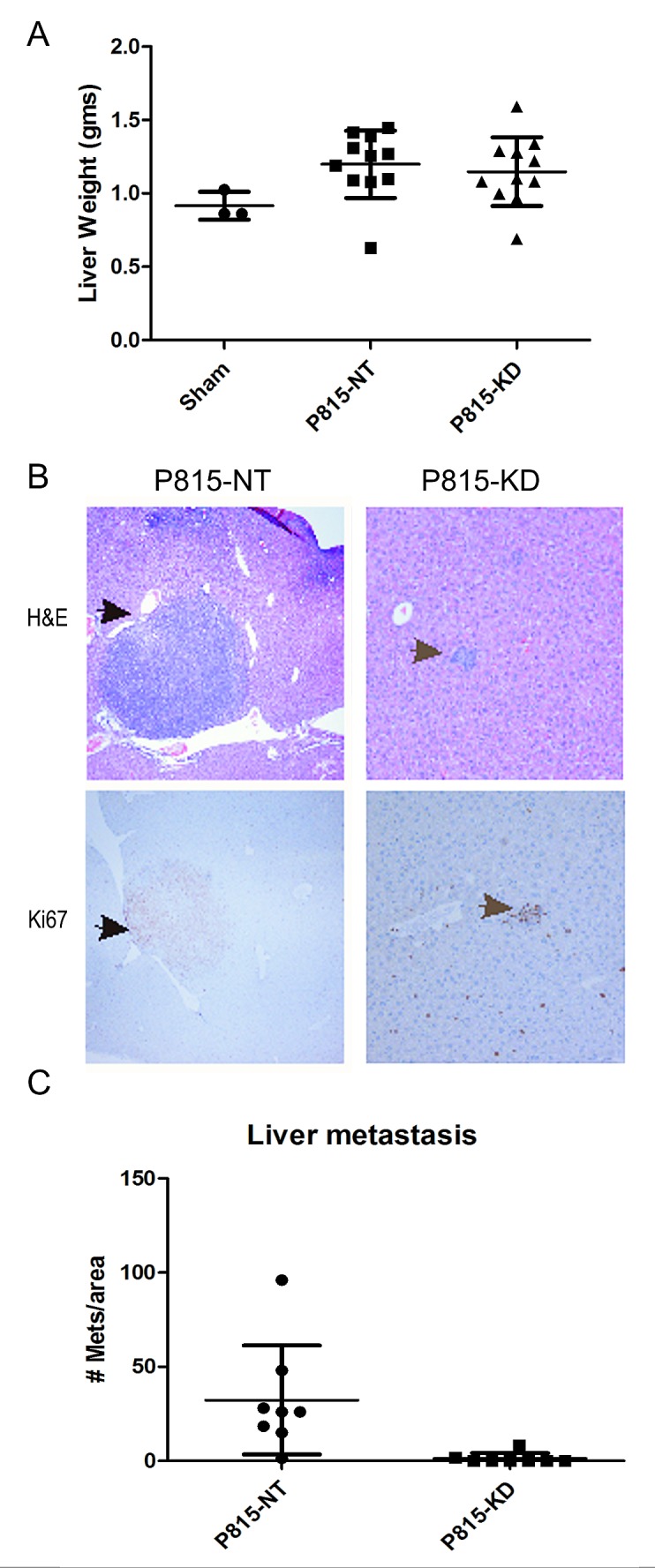
SHP2 silencing prevents mastocytoma tumor growth in liver A. Graph depicts day 13 liver weights from mice injected with saline (sham, n=3), P815-NT cells, or P815–KD cells (mean ± SD; n=11). B. Livers from mice injected as described above were formalin fixed, paraffin embedded, and sections were stained with H&E and by immunohistochemistry using Ki67 antisera. Representative micrographs are shown for H&E (upper panels) and Ki67 staining (lower panels; arrows denote mastocytomas and mitotic figures). C. Graph depicts quantification of liver metastases per unit area of liver section (mean ± SD; n=8 from 3 separate experiments; * indicates significant difference between NT and KD, p <0.05).

Since the liver was previously shown to be a site of P815 mastocytoma infiltration [34], we examined liver weight and cellularity between sham, P815-NT and P815-KD groups. Compared to sham controls, both P815-NT and P815-KD groups showed evidence of hepatomegaly (Fig. 5A). However, differences in liver weights between P815-NT and P815-KD groups were not significant (Fig. 5A). However, macroscopic nodules were observed on the surface of livers from the P815-NT group, but not the P815-KD or sham groups (data not shown). Furthermore, histological staining of liver sections from these mice revealed mastocytoma tumor nodules with clear, defined margins and an amorphous core (Fig. 5B, top panel). Metastatic cells in these tumors were poorly differentiated and the high nucleus/cytoplasm ratio suggested high mitotic activity, which was confirmed by immunohistochemical staining of Ki67 (Fig. 5B, lower panel). In contrast, livers from mice injected with P815-KD cells had only small cell aggregates resembling micro-metastases that were positive for Ki67 (Fig. 5B, right panels). However, no large tumors were detected in any of the P815-KD group (data not shown). Quantification of the mastocytoma burden in liver revealed a striking reduction in tumor incidence for the P815-KD group compared to P815 NT group (Fig. 5C). Taken together, these results implicate SHP2 in positively regulating progression of SM to aggressive, metastatic disease.

### SHP2 inhbitor II-B08 enhances the effects of Dasatinib on human and mouse mastocytoma cells

**Figure 6:**
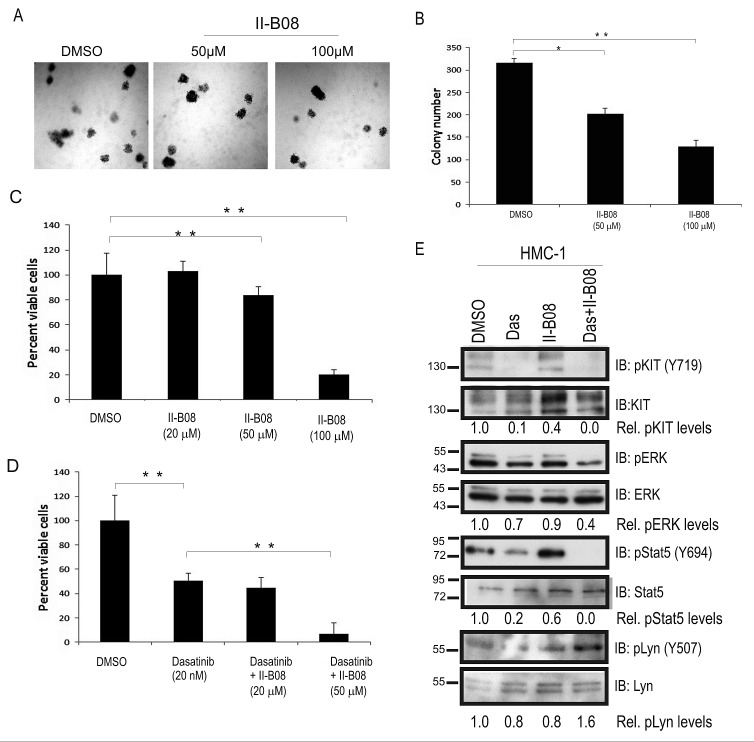
SHP2 phosphatase activity is required for sustaining growth and survival of HMC-1 cells A. Representative phase contrast images depicting the colony growth of HMC-1 cells in methyl cellulose media at day 8 in presence of DMSO and II-B08 (50 and 100 μM). B. Graph depicts average colony numbers (mean ± SD) for HMC-1 cells treated with DMSO or indicated concentrations of II-B08 in methyl cellulose medium at day 8 (triplicate samples; * and ** indicates significant difference between treatments at p<0.05 and p <0.01, respectively). C. HMC-1 cells were treated with DMSO or II-B08 (20, 50 or 100 μM) for 72h. Graph depicts the percentage of viable cells based on alamar blue assay (** indicates significant difference between DMSO and II-B08 treated cells, p <0.01). D. HMC-1 cells were treated with either DMSO, Dasatinib (50 nM), or Dasatinib (50 nM) plus II-B08 (20 or 50 μM) for 72h. Graph depicts the percentage of viable cells based on alamar blue assay (** indicates significant difference between treatment groups, p <0.01). E. HMC-1 cells were treated either with vehicle (DMSO), Dasatinib (5nM) or SHP2 inhibitor II-B08 (50 μM alone or Dasatinib (50nM) plus II-B08 (50 μM) for 4 h prior to lysate preparation and IB with pY719-Kit, Kit, pERK, ERK, pY694-Stat5 and Stat5, pY507-Lyn, and Lyn antisera. Densitometry values are shown below panels for relative phosphorylation levels (phospho-specific/total). Positions of relative mass markers in kilodalton are shown on left.

Previous studies have identified both phosphatase-independent and phosphatase-dependent roles for SHP2 in regulating cell growth and survival [35, 36]. To address this, we investigated the effects of a SHP2 phosphatase inhibitor (II-B08), which has shown anti-proliferative effects in several cancer models [30, 31, 33]. We employed the human mast cell leukemia cell line -1 (HMC-1) model, which harbors KIT D816V and V560G mutations, and depends on signaling from these constitutively active KIT receptors for growth and survival [4]. HMC-1 cells were allowed to grow in semisolid methyl cellulose media devoid of any colony stimulating factors, but supplemented with either DMSO or SHP2 inhibitor II-B08 (50 and 100 μM) for 8 days. We observed a dose dependent reduction in colony formation with II-B08 compared to DMSO control (Fig. 6A, B). These defects were likely due to loss of HMC-1 cell viability upon treatment with SHP2 inhibitor II-B08 as measured in Alamar blue assays (Fig. 6C). Since only the highest dose caused a substantial loss of viability, we considered combining II-B08 with other inhibitors. Previous studies have suggested that combination of drugs targeting different points in oncogenic pathways can improve neoplastic cell killing and the development of resistance [31, 37, 38]. To test whether SHP2 inhibitor could be effective in combination with multi-kinase inhibitor Dasatinib (BMS-354825), previously used in human mast cell leukemia SM patients [16, 37, 39], we treated HMC-1 cells with an IC_50_ dose of Dasatinib (50 nM) alone, or in combination with two different doses of II-B08. As expected, Dasatinib treatment alone caused approximately 50% reduction in HMC-1 cell viability compared to DMSO control (Fig. 6D). Interestingly, combination treatments with Dasatinib and II-B08 (50 μM) led to a significant improvement in HMC-1 cell killing compared to Dasatinib alone (Fig. 6D). Importantly, we observed similar results with II-B08 treatment of P815 mouse mastocytoma cells in vitro, and improved cell killing when combined with Dasatinib (20 nM) alone (Fig. S1). To assess the effects of this new combination treatment using II-B08 and Dasatinib on key cell growth and survival signaling pathways, HMC-1 cells were treated with either DMSO, Dasatinib (5 nM), II-B08 (50 μM) alone, or combined Dasatinib and II-B08 for 4h. Immunoblot analysis on cell lysates revealed that II-B08 alone had no effect on activation of KIT (Fig. 6E). While Dasatinib treatment impaired KIT phosphorylation dramatically, downstream signaling to Erk and Stat5 pathways were less severely impaired (Fig. 6E). However, combination treatments of Dasatinib and II-B08 caused significant reductions in Erk and Stat5 activation, along with increased inhibitory site phosphorylation of Lyn (Figure 6 e). Together with our results implicating SHP2 in promoting aggressive SM disease in mice, and the effects of SHP2 inhibitor treatment *in vitro*, we conclude that SHP2 inhibitors could be effective in combination with kinase inhibitors such as Dasatinib for treating aggressive SM.

## DISCUSSION

SM is an orphan disease with limited treatment options, mostly limited to treating adverse side effects of mediator release by neoplastic MCs [1]. Although several multi-kinase inhibitors can inhibit KIT^D816V^-driven growth of neoplastic MCs *in vitro* (midostaurin, ponatinib, sunitinib, Dasatinib), they have largely failed in clinical trials [13, 37, 38, 40, 41]. A phase II clinical trial of Dasatinib in patients with various myeloid disorders including SM, showed only partial response rates in SM (≈33%) associated with improved symptoms, but failed for patients with KIT^D816V^ mutations [14, 42]. The development of resistance to these kinase inhibitors also complicates the treatment strategies for SM, including emergence of other pathways (e.g. Stat5, Ras, SFKs, Tec/Btk kinases) that promote proliferation and survival independent of KIT^D816V^ in resistant tumors [18-20]. A recent study identifies combination treatments with multi-kinase inhibitors ponatinib and Dasatinib as more effective in blocking KIT^D816V^, Lyn, Stat5 and Btk signaling pathways [38]. Another potential target investigated here is SHP2 phosphatase, which has been identified as a druggable target in a KIT^D814V^-driven MPD mouse model [31]. Here, we show that SHP2 promotes growth and survival pathways in the P815 mouse mastocytoma model that harbors a KIT^D814Y^ driver mutation. Silencing of SHP2 impaired activation of ERK, Stat5, Lyn and Btk signaling pathways, and caused stabilization of the proapoptotic protein Bim. SHP2 KD cells showed defects in cell growth and increased apoptosis upon treatment with a KIT inhibitor *in vitro*. In a syngeneic mouse model of SM, SHP2 silencing led to a significantly reduced mastocytoma tumor burden and reduced metastases in the liver. Recent studies using SHP2 inhibitor II-B08 have provided evidence that SHP2 promotes proliferation and survival of leukemic cells harboring KIT and FLT3 mutations [31, 33]. We observed similar effects of II-B08 treatment (10-100 μM) leading to reduced colony formation ability of human and mouse mastocytoma cells (HMC-1 and P815). However, the effects of II-B08 on viability of these mastocytoma models were more pronounced when it was combined with Dasatinib. Mechanistically, this correlated with improved disruption of key cell growth and survival pathways (Erk, Stat5, Lyn) in Dasatinib/II-B08 treated HMC-1 cells compared to either drug alone. Overall, this study identifies SHP2 as a therapeutic target in aggressive SM which frequently harbor activating KIT mutations, and also provides further support for combining SHP2 and tyrosine kinase inhibitors in other relevant cancer models.

Several previous studies have implicated SHP2 in promoting wild-type KIT signaling to the Ras/ERK pathway leading to enhanced proliferation and survival of mature MCs [22, 27]. SHP2 also promotes activation of Lyn kinase and Stat5 pathways [31, 32], that are implicated in mechanisms to escape KIT inhibitor treatments [38]. In the current study, we also show that SHP2 promotes activation of Btk, which together with Lyn, can allow human MC leukemia cells to escape the effects of KIT inhibitors [18]. Thus, SHP2 inhibitors may be effective in targeting these resistant SM tumors, or more likely to be used in combination with multi-kinase inhibitors. In KIT^D814V^-driven MPD models, combination treatment with SHP2 and PI3K inhibitors was much more effective than single agent therapies [31]. Consistently, in this study we show that killing of mouse mastocytoma or human mast cell leukemia cells by submaximal doses of Dasatinib, is improved upon the addition of SHP2 inhibitor II-B08 in *in vitro* assays. The rapid development of ASM in the syngeneic model used here, should allow for future testing of existing or new SHP2 inhibitors in single or combination therapies in future

To fully understand the contributions of SHP2 to SM progression in vivo, the potential contribution of SHP2 to the homing of neoplastic MCs to various organs should be investigated. This is partly due to a recent study showing that SHP2 KO HSCs are defective in homing to BM in irradiated mice [24]. Thus, the more dramatic defects of SHP2 silencing that we observed in the in vivo model compared to the in vitro assays, may reflect contributions of SHP2 to both growth/survival signaling and homing of mastocytoma cells. Another possible explanation comes from a recent study implicating SHP2 in maintaining a pool of breast tumor-initiating cells via a c-Myc/ZEB1-dependent gene expression signature [43]. It will be interesting to test for involvement of this putative SHP2 gene signature in the context of mastocytoma tumor-initiating cells and potential mastocytoma stem cell populations. In addition, the recent description of a Cre/LoxP system that controls KIT^D814V^ expression [9] will allow for coordinated KIT^D814V^ expression and SHP2 KO using these transgenic mouse models. We previously used Mcpt5-Cre to generate MC-specific SHP2 KO mice, and observed a significant loss of connective tissue MCs [22]. Given that bitransgenic Mcpt5-Cre:KIT^D814V^ mice develop SM [9], it will be interesting to test the effects of SHP2 KO on disease onset, and progression in this model. This transgenic model can also be employed for further testing of combination treatments with SHP2 and KIT inhibitors in vivo.

In conclusion, our study identifies SHP2 as a vital effector of oncogenic KIT signaling in a model of aggressive SM. Further, we demonstrate that SHP2 inhibitors can be effective in combination with multi-kinase inhibitor Dasatinib in killing human and mouse mastocytoma cells *in vitro*. Future testing of SHP2 inhibitors, including novel inhibitors with lower IC_50_ values, will undoubtedly spur further testing of multi-class inhibitors against oncogenic kinases and phosphatases for the treatment of aggressive SM and other cancers.

## MATERIALS AND METHODS

### Cell lines and reagents

P815 cells dervied from mouse mastocytoma were purchased from ATCC (VA, USA) and maintained in complete Dulbecco's modified Eagle's medium (DMEM) supplemented with 10% fetal bovine serum (FBS) at 37 ºC in 5% CO2 incubator. HMC-1 cells were kindly provided by Dr. Joseph Butterfield (Mayo Clinic, MN),[4] and cultured as above. The following primary antibodies were used: rabbit anti-Bim (C34C5; 1:1000; Cell Signaling Technology (CST)), rabbit anti-phospho-Bim (Ser69; 1:1000; CST), rabbit anti-phospho-Btk (Tyr 223; 1:1000; CST), rabbit anti-Btk (D3H5; 1:1000; CST), rabbit anti-phospho-Stat5 (Y694; C71E5; 1:1000; CST), rabbit anti-Stat5 (1:1000; CST), mouse anti-phospho-ERK1/2 (E4; 1:1000; Santa Cruz Biotech Inc. (SCBT)), rabbit anti-ERK (K23; 1:1000; SCBT), rabbit anti-SHP2 (C18; 1:1000; SCBT), rabbit anti-Lyn (SC-44; 1:1000; SCBT), rabbit anti-phospho-Lyn (Y507;1:1000; Epitomics), rabbit anti-phospho-KIT (Y719; 1:1000; CST), and rabbit anti-KIT (1:1000; CST). Other reagents included: horseradish peroxidase-conjugated goat anti-mouse IgG and anti-rabbit IgG (GE Healthcare), enhanced chemiluminescence reagent (Perkin Elmer), Dasatinib (LC laboratories), SU11652 (Calbiochem), and the previously described SHP2 inhibitor II-B08.[30] Antibodies for flow cytometry included: PE-conjugated anti-mouse CD117 (2B8; BD Bioscience), PE/cy5-conjugated anti-mouse CD45 (eBioscience), PE conjugated anti-mouse CD11b (M1/70; Biolegend), PE/cy5-conjugated anti-mouse CD45R (B220) (RA3-6B2; Biolegend), FITC anti-mouse Gr1 (RB6-8C5; Cedarlane).

### Lentiviral transduction of P815 cells

pGIPZ-puro vector expressing shRNAs targeting SHP2 (shRNA1: clone C2 (V2LMM_782273), mature antisense ATATTGGTATATTCATGTC; shRNA2: clone F11 (V3LMM_430010), mature antisense TGAGCTCGATAACATCTCC)), and a non-targeting (NT) shRNA control from Open Biosystems. Lentiviral production and transduction were performed as previously described [22]. Briefly, lentiviral supernatants prepared with shRNA against SHP2 and NT control were incubated with P815 cells for 48 h and followed by selection with puromycin (500 ng/ml) for 5 days. Stable cell pools were screened for the most effective shRNAs clones via subjecting lysates from repeated passages to immunoblot (IB) analysis.

### Cell viability assays

P815 cells or HMC-1 cells (1×104 /well) were incubated with DMSO or IIB 08 (20-100 μM) alone or in combination with Dasatinib with indicated concentrations for 72 h in a 96 well plate. Alamar blue dye (10 μL) was added to each well for 2 h prior to analysis using a spectrophotometer as described previously [22]. All optical density (OD) readings were normalized against that of DMSO treated P815 cells or HMC-1 cells. To calculate percent viable cells, a standard curve was included with known cell numbers for either P815 or HMC-1 cells at time the time of Alamar blue addition.

### Cell lysate preparation

P815-NT, -KD1 and -KD2 (5 × 10^6^) cells were plated in serum free DMEM for 2 h followed by replacement with complete DMEM for 1h. Soluble cell lysates (SCL) were prepared by incubating cells in ice cold Magnesium lysis buffer (50 mM Tris-HCL-pH 7.5, 150mM NaCl, 1% (v/v) Triton X-100, 0.25% Sodium deoxycholate (w/v),10% glycerol, 25 mM NaF, 10 mM MgCl2, 5 mM EDTA, 10 μg aprotinin/mL, 10 μg leupeptin/mL, 1 mM vanadate, 100 μM phenylmethyl sulfonyl fluoride) for 15 min and followed by centrifugation at 12,000 g for 15 min. In inhibitor studies, HMC-1 cells were plated in complete DMEM and treated for 4h with either DMSO, Dasatinib (50nM), II-B08 (50 μM) alone or in combination of Dasatinib and II-B08 and cell lysates were prepared as described above.

### Cell proliferation, colony, and apoptosis assays

P815-NT, P815-KD1 and P815-KD2 (1 × 10^5^) cells were plated in complete DMEM in triplicate wells, and total cell numbers were scored at 24, 48 and 72 hours using an automated cell counter (Beckman Coulter Z2 particle analyzer). For colony formation assays, P815-NT and P815-KD2 (2 × 10^4^) cells were suspended in MethoCult media (M3231; Stem Cell Technologies) and plated in a 35 mm sterile tissue culture plate as per manufacturer's instructions. Colonies were manually counted after 8 days by phase contrast microscopy (Nikon Eclipse TS 100 light microscope). For inhibitor studies, HMC-1 or P815 (2 × 10^4^) cell were plated with DMSO or II-B08 with indicated concentrations with further addition of same doses at 72 h to maintain inhibitor concentration throughout experiments. Images were acquired and colonies were counted at day 8. P815-NT and P815-KD2 were incubated with DMSO or SU11652 (250 nM) for 18 hours, stained with FITC-AnnexinV and propidium iodide (PI). Apoptotic cells were identified as AnnexinV^+^PI^−^ cells by flow cytometry (gating was based on unstained cells).

### Mouse model of aggressive SM

DBA/2 mice (6-8 week old) were purchased from Charles River Laboratories (Raleigh, NC, USA), and housed at Queen's Animal Care Services according to protocols approved by the Queen's University Animal Care committee in accordance with Canadian Council on Animal Care guidelines. P815-NT or -KD2 (5 × 10^3^/100 μl of PBS) were injected in retro-orbital sinus using 27 gauge needle under anesthesia (isoflurane). Mice had become moribund by day 13-16, and were anesthetized prior to cardiac puncture, and sacrificed prior to collection of bone marrow, spleen and liver tissues. Single cell suspensions were obtained from bone marrow, blood and spleen prior to red blood cell (RBC) lysis and staining with PE-conjugated anti-mouse CD117, PEcy5-conjugated anti-mouse CD45 to identify mastocytoma cells. PE-conjugated anti-mouse CD11b, PEcy5-conjugated anti-mouse CD45R (B220) and FITC-conjugated anti-mouse Gr1 staining were used to identify macrophages, B cells and neutrophils, respectively. All samples were analysed by flow cytometry, with gating set using isotype control antibodies. Subsequent data analysis and histogram preparation was performed using Flowjo software. Absolute numbers of cells were calculated by multiplication of total cells obtained from the target organ and percentage population determined by flow cytometry for indicated immune cell markers. Liver tissue sections were analyzed by hematoxylin and eosin (H&E) staining and metastatic tumors were quantified from 5 randomly selected fields of view.

### Statistical analyses

Differences between P815-NT and P815-KD cells were tested using paired student T-test, and significance differences were indicated by *P* <0.05 (Microsoft Excel).

## SUPPLEMENTARY MATERIAL AND FIGURE


