# Supplementary material for: Oncogenic KIT-induced aggressive systemic mastocytosis requires SHP2/PTPN11 phosphatase for disease progression in mice
Source: Oncotarget. 2014 Jul 7;5(15):6130–41. doi: 10.18632/oncotarget.2177 (PMC4171618; doi:10.18632/oncotarget.2177)
Supplement: Supplementary file 1 [file oncotarget-05-6130-s001.pdf]

Oncogenic KIT-induced aggressive systemic mastocytosis requires SHP2/PTPN11 phosphatase for disease progression in mice

SUPPLEMENTARY MATERIAL

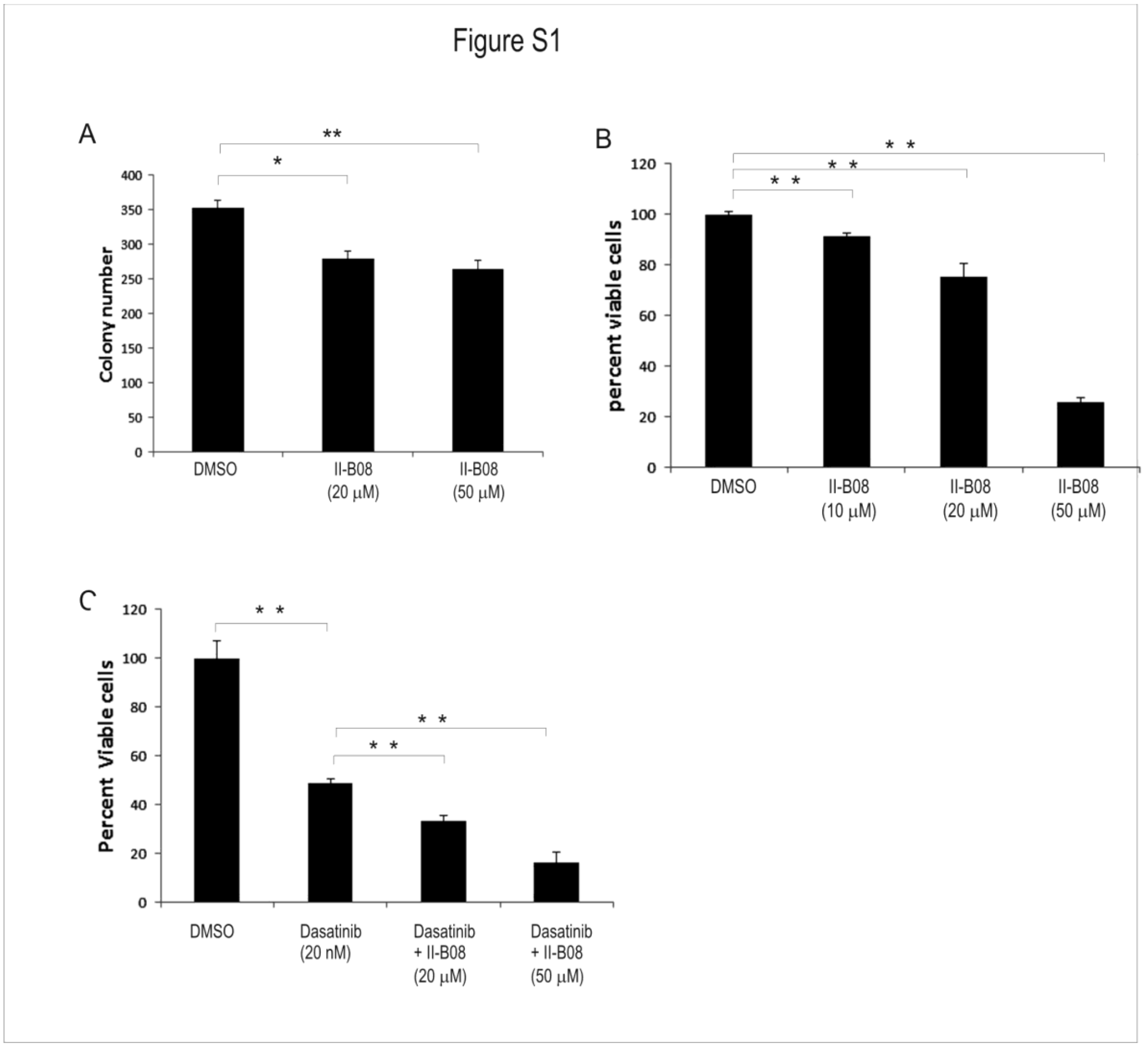

Fig. S1: SHP2 inhibitor treatment blocks growth and survival of mouse mastocytoma cells. A. Graph depicts average colony numbers (mean ± SD) for P815 cells treated with DMSO or indicated concentrations of II-B08 in methyl cellulose medium at day 8 (triplicate samples; * and ** indicates significant difference between treatments at $p<0.05$ and $p<0.01$, respectively). B. P815 cells were treated with DMSO or II-B08 (10, 20 or 50 µM) for 72h. Graph depicts the percentage of viable cells based on alamar blue assay (** indicates significant difference between DMSO and II-B08 treated cells, $p<0.01$). C. P815 cells were treated with either DMSO, Dasatinib (20 nM), or Dasatinib (20 nM) plus II-B08 (20 or 50 µM) for 72hours. Graph depicts the percentage of viable cells based on alamar blue assay (** indicates significant difference between treatment groups, $p<0.01$).
